# Respecifying social change: the obsolescence of practices and the transience of technology

**DOI:** 10.3389/fsoc.2023.1222734

**Published:** 2023-10-03

**Authors:** Jakub Mlynář, Ilkka Arminen

**Affiliations:** ^1^Institute of Informatics, HES-SO Valais-Wallis University of Applied Sciences Western Switzerland, Sierre, Switzerland; ^2^Faculty of Social Sciences, University of Helsinki, Helsinki, Finland

**Keywords:** ethnomethodology and conversation analysis, history, social change, sociology, temporality, technology, landline and mobile telephony

## Abstract

This article proposes that social change, a fundamental topic in sociological theory, can be productively revisited by attending to studies in ethnomethodology and conversation analysis (EM/CA). We argue that the corpus of EM/CA research, from the 1960s until the present day, provides details of the constitutive and identifying aspects of practices and activities that gradually transform into descriptions of *obsolescent* practices and activities, and that this corpus can be revisited to learn about the ways people used to do things. Taking landline and mobile telephony as a case in point, we show that the subtle details of conversational practices are anchored in the technology used as part of the contemporary lifeworld, and that they stand for the particularities of routine social structures of their time period. We also discuss the temporal aspects of the competences required on the part of members and analysts to make sense of encountered practices in terms of their ordinary recognizability and interactional consequentiality, pointing to the anchoring of social life in its historical time. Finally, we conclude by considering different ways of respecifying social change by attending to various kinds of historicity and obsolescence of social praxis.

## 1. Introduction

In a 2005 paper on “maps and journeys,” Brown and Laurier offer a detailed description of travelers’ work with a paper map as part of a car journey. Among other aspects of the activity, the authors take into account the positioning of the map as a material object incorporated in the social activities: “When closed, it lies on Jane’s lap, and although she opens up the map and makes it available to Fay (who uses it to point at), she does not move the map to the middle between them. … Confirmations of what they are seeing in common are marked by gestures: they point at features, bring out routes, and are otherwise immersed in the tangle of marked roads on the map, with points and sustained followings of their fingers. Because they are doing this naming and pointing together, should Jane make a mistake, Fay can correct her, and vice versa” (Brown and Laurier, 2005, p. 27). Although the analytic account is poignant and careful, the described activity might strike a current reader (i.e., in 2023) as somewhat dated, given the transition from paper maps to digital navigation devices. This becomes apparent when one compares Brown and Laurier’s analysis with a more recent description of “navigating with digital maps” provided 15 years later by Smith et al. (2020, p. 229): “During Bryn’s questions, Aled glances at the screen of his mobile device, maintaining the relevance of the WWR app^1^ as the basis for restating his proposal, ‘I think we (.) carry on’ […] when Aled pauses […] he raises a pointing finger to the device’s screen and, at the same time, rotates it towards Bryn and steps slightly backwards as Bryn closes in. His adjustment of the device angle enables both of them to see their current location on the app and the suggested routes to the Roman camp. This deft set of movements supports co-viewing of the smartphone’s screen, while simultaneously making it relevant to the current navigational trouble.” Although the participants in both instances are involved in a similar mundane activity of wayfinding with a map, their social practices, material tools, and routine ways of working—preserved and represented in the quoted descriptions and in the remainder of the two papers—are significantly different. Such noticeable transformations in everyday and professional activities over time provide grounds for the main arguments of the present paper.

Social change is one of the central and perennial topics of sociology and the social sciences (Sztompka, 2000; McLeod and Thomson, 2009). The very foundations of the discipline rest on the recognition of profound transformations in the established common ways of life, experienced from the eighteenth and nineteenth centuries with the onset of industrialization, urbanization, and the related emergence of “modernity” (Ballard and Barnett, 2023). Narrative conceptualizations of history emerged in Europe around the same time (White, 1973; Koselleck, 2004), since a reflexive historicity is a cornerstone of modern society that—as a “self-describing object” (Luhmann, 1992)—also produces accounts of itself with regard to collective pasts and futures. Such reflections of social change are often connected to its assessment, applying and variously favoring conceptions of progress, decline, or continuity (Weeks, 2007). May (2011, p. 367) points out that “a focus on the everyday allows us to view social change not simply as a top-down process generated by ‘extraordinary’ events but as something that also results from our mundane ‘ordinary’ activities.” Aligning with her suggestion, this article extends an invitation to scholars in the social sciences to consider research in ethnomethodology and conversation analysis (henceforth EM/CA)^2^ as offering a distinct and valuable historical perspective, although the studies are rarely conceived or conducted as investigation of history or social change as such (Lynch, 2009; Pekarek Doehler et al., 2018).

Our aim is twofold: first, to outline a praxiological respecification of “social change” as a focal topic of the social sciences, and, second, to offer a novel look at the corpus of studies of practical action and practical reasoning collected within EM/CA. These studies address the lived interactional present and the endogenous time of locally organized social settings, explicating the ways in which recognizable scenes of everyday life are produced. We argue that in doing so, EM/CA research also inevitably and unavoidably—though mostly inadvertently—provides accounts of practices that are reflexively entrenched in the exogenous time of social processes. *First*, with regard to the respecification of social change, we develop EM’s central strategy: “while taking up recognizable topics in philosophy and social theory, ethnomethodology makes a deflationary move to respecify them praxiologically” (Lynch, 2022). As Button points out, EM (and CA) is interested in foundational sociological matters in an alternate way: “it wished to make them investigatable, available for enquiry. In holding them up for scrutiny, and in working through the implications of that enquiry, ethnomethodology came to respecify foundational matters” (Button, 1991, p. 5). We argue that research in EM/CA, viewed in retrospect, makes social change as a foundational matter of social science visible and investigable. Therefore, we aim at articulating some blind spots of theories of social change (e.g., Eisenstadt, 1964), and provide a distinct perspective *vis-à-vis* more recent mid-level conceptions on technology-related social change in Science and Technology Studies (e.g., Sørensen, 2006; Wyatt, 2008). Relatedly, regarding our *second* aim, we propose that the corpus of EM/CA studies can be conceived as offering a distinct historical perspective on society. From EM/CA’s corpus of empirical studies, gathered over more than 60 years, we learn not only about “how people actually do things” (Livingston, 2008, p. 842) but also how people *used to* actually do things, as practices that were once unproblematic and taken for granted gradually become outdated.

Commenting on an assignment he gave to his students in 1960s on observing people as they are “exchanging glances,” Harvey Sacks (1992, Vol. I, p. 94, emphasis added) also contemplated the historical dimension of everyday life: “I know that people can do this, I’ve watched it many times, and I take it that you have seen it also. … [But] it could have been the case that everybody came back and said ‘No, I never saw that happen.’ And that’s possible. It might be *something that’s dying out*. *A thing that our forefathers had*. Like God.” To grasp this inherently and inevitably transient character of social praxis in current societies, this article introduces, lays out, and illustrates the notion of *obsolescence*. Findings of EM/CA become obsolescent in the sense that they capture particulars of social practices in terms of their constitutive and identifying details, but at the same time these described particulars always consist of things of the past, and they might comprise former ways of life that are no longer to be found in the world. Once social practices are encountered—documented *in vivo* or in published literature—as obsolete, one encounters social change as an aspect of everyday life, ingrained in its details.

Reflections of social change in scientific and everyday discourses are often tied to technological development (White, 1962; Bittner, 1983; Button, 1993), and sociology considers technology both as “an agent and an object of social change” (Kinsley, 2023, p. 250). In this paper, we also approach the theme of social change through a focus on how technological objects are “made at home in the world that has whatever organization it already has” (Sacks, 1992, Vol. II, p. 549). In resonance with the proposition of Deppermann and Pekarek Doehler (2021, p. 131), our case here is telephony: the first machine-mediated synchronous interpersonal exchanges, which are themselves a novelty in human history. Through secondary analysis of materials from CA studies on landline and mobile phones, in the following two sections we provide grounds for the introduction and explication of the notion of obsolescence. Subsequently, in the remainder of the text, we conceptually respecify this notion from an EM perspective, reflecting on how such empirical materials can be “made sense of” as documents of the past by both members and analysts.

## 2. Mobile and landline telephony: emerging obsolescence

Modern telephony was born and subsequently evolved quickly in the latter part of the nineteenth century through a series of innovations that led to telephone calls being transmitted with lines, thereby earning them the name “landline telephone,” which continued as the reigning form of telephony for the next 100 years. Though within “landline telephony” there were several steps of development, such as the automation of switchboards (which made the “central ladies” redundant), telephone etiquettes evolved and were standardized in a step-by-step manner in varying national and linguistic contexts. One aspect of the evolving telephone etiquette was how to answer and open the conversation (Hopper, 1992). Following Schegloff (1968), there emerged within CA a tradition of telephone conversation opening studies, which demonstrated the local patterns and regularities of openings in a number of countries and languages (e.g., Houtkoop-Steenstra, 1991; Hakulinen, 1993; Lindström, 1994). It appears that in the course of the development of telephony, call openings had been conventionalized and highly standardized, involving strong regularities but also a linguistic and cultural sensitivity (Arminen and Leinonen, 2006).

For instance, the Finnish opening pattern of landline calls had become robustly canonized. It can be estimated that well over 90% of calls had the same pattern (Arminen and Leinonen, 2006). Finnish calls were opened with a self-identification that was an answer to a summons, which in landline telephony was a telephone ring summoning hearers to respond by picking up the receiver. Canonically, the answerer’s first turn received a reciprocal self-identification from the caller, which followed a greeting. After the return of the greeting, the call was ripe for the initiation of the topic of the call. Excerpt 1 below presents a case in point (C = caller; R = answerer; transcription conventions are explained in the appendix; data is from the Finnish Department Data archive, University of Helsinki, Finland).

**Excerpt 1:**
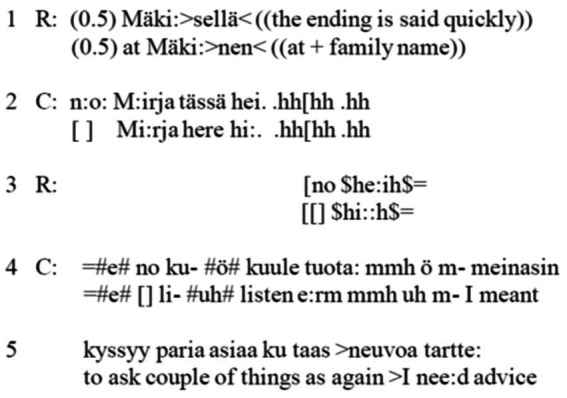
(Arminen and Leinonen, 2006, p. 342).

For our purposes, the first line is the crux of the matter. First of all, there is a pause in the beginning, and an inpatient reader might doubt the accuracy of the notation. Notably, though, the landline calls were opened when the call recipient picked up the telephone receiver (though there was some variation in the design of telephone apparatuses). As the recordings of calls were set to capture the whole call from the opening of the line to its closing, there tended to be a brief moment—not really a silence, but a low noise marking the connection made on the line, presumably standing for the moment when the answerer had picked up the receiver, opening the line. In the landline call opening, the line-opening sound^3^ was part of the opening, indicating that the receiver had been picked up and the answerer was about to speak; in this way, the initial pause belongs to the answerer, as transcribed here. It also stands for the technical possibilities and limitations of landline telephony.

The linguistic content of the rest of the first line includes a self-identification that can notably vary. Here, the answerer utters a family name and a case marking that indicates location. The opening thus displays the call to have reached a certain family at their location. In that way, this opening line, which is not atypical, is also in this part indexical to the type of technology used; that is, the landline calls were connected between points in the telephone network, and here the speaker vocalizes their spatial point in the network. Furthermore, the use of family name indicates that the telephone belonged to the family. It also opened varying trajectories for the call, depending on who would turn out to be the intended recipient. Hence, the opening was indexical both to the particularity of technology in its time and to the particularities of social formation, revealing that the technology use was not individual but based on units that shared a telephone, such as families or offices.^4^ Thirdly, the answerer’s first line did not show orientation to the caller’s identity. That is, the analogue telephone ring—the summons—did not carry information of who the caller was. Given the anonymity of the summons, the answerer had to respond without knowing who the caller was or what the reason of the call was. This lack of knowledge was imprinted in the analogue landline call openings, irrespective of whether they were based on self-identification, as in numerous countries in Europe, or included a voice sample, as in Anglo Saxon countries (Arminen, 2005). The lack of knowledge of the caller and of the call topic is hugely salient in that it shows that the parties on the phone lacked a connection and awareness of those who were outside of the proximity of their own location. Though this may not appear much of an observation, it pinpoints a significant aspect of the lived life of its time.

It is also notable that in the era of landline telephony there appeared aspirations to reach beyond the limits of the horizon of the moment. Garfinkel reflected on these aspirations through a tutorial on telephone summons, where he asked his students to tape record a phone ringing that is audibly summoning them, or someone else, or nobody in particular, etc. (Garfinkel and Wieder, 1992).^5^ Schegloff (1986) also paid attention to the answerer’s potential orientation to knowing who is calling. Mostly, answerers gave a voice sample “hello?,” which did not display knowledge of the caller’s identity; the answerers could also greet the caller with “hi,” displaying their “super-confidence” (Schegloff’s term) in who was calling.^6^ In this way, the explication of the lived practice of the time discloses correlations with socio-technical historical moments. Notably, both Garfinkel and Schegloff in their studies on telephone summons traded on the technology of its time and exposed the technology users’ taken-for-granted assumptions of that world. Bjelić (2019) even suggests that call recipients demonstrated a particular capability to orient a telephone ring to be from a particular caller, which stands for the lifeworld of landline call recipients. Following Sacks, we may say that here the EM/CA studies have articulated a historical moment of the way how preceding generations have acted (up to the 1980s).

## 3. The vanishing lifeworld of landline telephony

Landline telephony stood for the lived world where remote communication took place between designated fixed points. This required practices that parties used for communication between the points when telephony was not available. As a case in point, a childhood recollection of one of the authors (JM) captures the life lived in-between the telephone network points:

*Growing up in Central Europe in the early 1990s, I remember that we spent a lot of time playing outside with other kids from the neighborhood, in the concrete streets of the housing development. While spending an afternoon with friends away from home, kids usually had the duty of “reporting themselves,” for the parents to know that their child is all right. I remember that we did this by ringing the doorbell and saying through the speaker something along the lines of “I am just reporting myself,” and the parent usually specified that you had to come back at a certain time, typically for dinner, or maybe come home immediately and do your homework. This practice was, as I remember it, common and mundane. Most of us did it and we gave it no second thought as we often accompanied each other for such a quick “reporting” at home*.

All these practices underwent profound changes when mobile telephony emerged.^7^ Wireless technologies started to quickly evolve in the 1970s and 1980s, and their standardized forms diffused at a record-fast speed in the 1990s, largely replacing landline telephony. Mobile telephony led to numerous changes in phone calls that could be traced already in the openings (Weilenmann and Leuchovius, 2004; Arminen and Leinonen, 2006; Laursen and Szymanski, 2013). Mobile call openings typically resemble what Schegloff (1986) called super-confident landline call openings. Most mobile phones, being based on digitized telephone systems, allow the receiver to gain access to the caller’s number so that the answerer may know who is calling before answering the call. If the caller’s number is listed on the answerer’s mobile phone contacts list, then the caller’s name may appear or a personalized ringtone may sound. Consequently, the answerer—for a good reason—can be super-confident about who is calling (though only if the call comes from the listed number). The answerer thus may tailor their answer accordingly, as seen in the excerpt below (T = caller; S = answerer; data archived, IA, University of Helsinki, Finland) (Excerpt 2).

**Excerpt 2:**
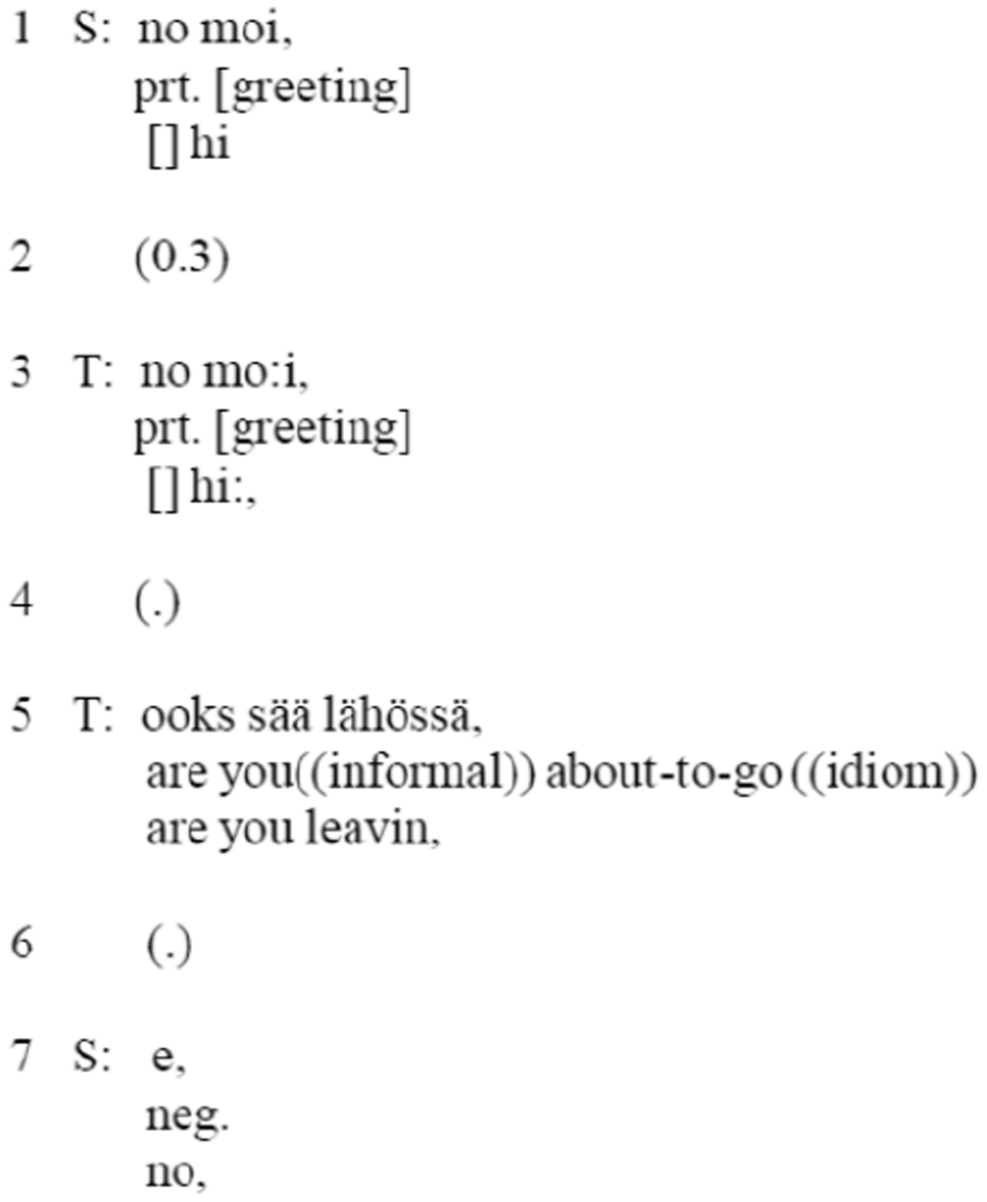
(Arminen, 2005, p. 651).

In comparison to a landline call opening, we can notice several distinctive features here. First, the ubiquitous mobile technology is individualized, compared to landline telephones that were shared with others. In Finnish mobile calls, it is not uncommon to start the answer to the summons with a speech particle “no.” Basically, the Finnish “no” is untranslatable, at least into English,^8^ but it is a speech particle that is both backward- and forward-looking. In other words, through “no” the answerer gives the answer as having been responsive to a recognized action and also initiates a transition to a new stage in the conversation. A reader may pay attention to the fact that in the landline call the same particle was initially used by the caller notifying the answer and initiating the next move [see (1) line 2]. It appears that this shift is a systematic change toward a novel social practice (Arminen, 2005; Arminen and Leinonen, 2006). In this way, in mobile calls the answer to the summons that allows the recipient to get to know who is calling is designed as a move for an already ongoing interaction. Unlike landline calls, the recipient design of the call begins already in the answer to the summons, which makes a recipient-designed response relevant. Reciprocally, the caller may also assume who is likely to answer, as mobile phones are personal, unlike collective landline phones. Consequently, the greeting exchange happens between parties who know each other, and there is no need for identification work, voice samples, or self-identifications. After the exchange of greetings, the anchor position for the reason for the call is established (line 5). The opening is thus systematically truncated in comparison to analogue landline openings.

Nevertheless, as with landline telephony, the subtle details of the conversational practice correlate to the technology used; in that way, they also stand for the particularities of the routine social structures of their time. Mobile telephones are wireless and miniaturized, allowing ubiquitous communication. Already in the opening sequence, the participants display their reciprocal identification of each other and the immediate readiness to move to discuss their current activities, arrangements, and locations. That is, the epistemic ecosystem of telephones has undergone a profound change, from analogue landlines to digital mobiles. The resulting outcome could be called the lifeworld of “connected presence” (Licoppe, 2004). Ubiquitous communication technologies enable social exchanges between people beyond the bounds of time and location; no less importantly, they merge mediated and co-present relations, forming a presence that is connected to online realms beyond the immediate moment. Compared to the lifeworld of landline telephony, the pervasive communicative access between individuals incorporates offline and online environments, making contact potentially ceaseless and all-encompassing and also transforming family practices from the previous era of communication between the points (Lahikainen and Arminen, 2017).

## 4. The analytic relevance of obsolescence

The analytic relevance of obsolescence can be demonstrated with the help of a case in which it has been missed. That is, EM/CA studies do not automatically guarantee a sensitivity to historical changes, for to be alert to emerging obsolescence requires scholarly expertise. Empirically, analysis must be rigorous and strict to reveal the changes in interactional practices that have made some aspects of the former practice obsolete. Without sufficient understanding of the former interactional practice, the analyst may not be able to apprehend the relevancy of details that have replaced some of its aspects. The changes in interactional practices are also related to and comprise a consequential part of the historical alteration of lifeworlds.

In their comparison of landline and mobile call openings, Hutchby and Barnett (2005, p. 147) stated that “far from revolutionizing the organization of telephone conversation, mobile phone talk retains many of the norms associated with landline phone talk.” Using our terminology, for these authors the landline calls, their associated norms, and the lifeworld based on communications between the network points had not become obsolete. To make their point, they demonstrate the structure of mobile call openings, starting from the extract below (Excerpt 3). In line 3, the answerer responds to the summons (lines 1–2) and receives “how are you” (line 3), after which the answerer makes the first initiation for the topical talk (line 4; SB = answerer, Irene = caller).

**Excerpt 3:**
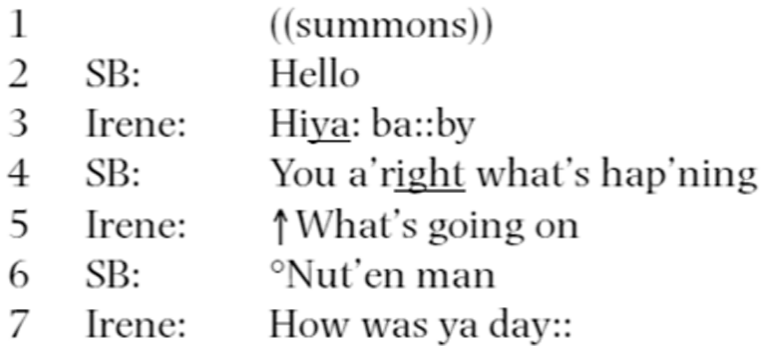
(Hutchby and Barnett, 2005).

To defend the all-encompassing power of the landline calls and their lifeworld, Hutchby and Barnett (2005, p. 157) state that in the mobile call openings there appears to be nothing “mobile”; if there are changes to landline call openings, these changes are not pervasive, but just “subtle details of the organization of interaction.” There appears to be at least six subtle details in these openings that stand apart from the landline call openings (Arminen, 2005): These include: (1) Answering a mobile phone summons differs prosodically from the answers to summons of landline telephones. The Anglo Saxon landline answers to the summons “H’llo?” were typically produced with a rising intonation (marked with ‘?’), which Schegloff (1968) calls a voice signature. In Hutchby and Barnett’s data (or any other mobile phone data), there is no trace of voice signature prosody. (2) In landline calls, the answer to the summons is not a greeting, and the greeting exchange follows it, but that is not the case in mobile call openings. (3) In landline calls, either the answerer has to identify the caller or the caller has to identify themself. In mobile calls, the conversational identification work has largely become obsolete for the caller, as the digital mobile system provides caller identification. There is a conversational work of recognition, but no work of identification (see also Button et al., 2022, p. 88).^9^ (4) In landline calls, the answerer and caller display a reciprocal recognition before the topic initiation. In mobile calls, this recognition work is found already in the call opening. The flat “hello” works as a greeting and is responded to with a greeting conveying the recognition of the caller, both in English (Excerpt 3) and Finnish (Excerpt 2) examples. (5) The landline calls were made between the spatial points of the network, which made it relevant for the caller to disambiguate whether the right person in the network had been reached. Parties in ubiquitous individualized mobile telephony are relieved from the disambiguation task. (6) Due to all the aspects above, the opening sequences of mobile calls became systematically radically reduced compared to landline telephone opening sequences. This does not mean that there was no perseverance of interactional practices between landline and mobile call openings. Exchanges of greetings (both in Finnish and English data) and how-are-yous (in English data, as previously) do take place, but technologically afforded identification and ubiquity of calls have enabled the emergence of a set of new practices, as listed above, amounting to the obsolescence of a lifeworld of communication between network points.

At this point we can formulate some preliminary conclusions. First, the “subtle details of call openings” are part of the complex orchestration of intersubjectivity. If we fail to pay attention to these, we risk also missing the achieved sense of action in interaction, and we may not grasp the relevance and consequentiality of the action. Second, the analysts’ action ascriptions are consequential. If we state that there is no salient difference between landline and mobile telephony, we also claim that no significant social change has happened. When there is no social change, there is also no obsolescence. The world in which there is no history—or social change, or differences between historically altered social practices—is a world where all cats are grey. Researchers need to carefully attend to elaborate details of practical action, while articulating the lifeworld contextures of the described practices and their inevitable embeddedness in sociohistorical environments.

## 5. Grasping the past: historical unique adequacy

Our comparison of routine practices in landline and mobile telephony has shown that a social change can be made visible as a contrast between the past and the present. If a researcher is interested in social change, then the focus will be on novelties in social conduct, though continuities may also exist. It is the intertwining of familiar and strange, the tension between the surprising and the well known, which provides for the visibility of social change in everyday praxis. The ability to see a practice as *obsolescent* (or, conversely, as *contemporary*) opens a possibility to grasp its historicity, but that is not a taken-for-granted competence. When a person encounters something that one has never seen happening (e.g., in an old movie) and is unable to understand what is going on, the experience as such does not open a vision of history and social change. One needs to have sufficient practical or theoretical expertise to recognize a practice for what it is, and only afterwards is one able to articulate and disambiguate the embeddedness of the practice to its sociohistorical environments, beginning to see a society with a history.

Encountering empirical materials from former times, such as writings, photos, audio, or video recordings, requires an ability to grasp and understand the social practices that are captured in these materials. Phenomenal features of social activities can be *preserved* for recognition and analysis (Mondada, 2006), but it is always necessary for the analyst to be able to make sense of them. Essentially, the analyst is dealing with the problem of retrospective sense-making in terms of “actors” that are divided from them by the passage of time. A certain bit of conduct that was a recognizable social practice in the past may lose this recognizability, and just how it is consequential in a particular moment of interaction becomes lost. This raises interesting questions about the possibility of “intersubjective understanding” across extended periods of time, and about building coordinated social action with materials provided by temporally distant actors as predecessors (see Schutz, 1967; Goodwin, 2018). The concept of the past depends on the relevance of the past for the present “here and now.” A praxiological respecification of this central element of social change is related to a consideration of the historical dimension of *the unique adequacy requirement of methods*.

In ethnomethodology, the unique adequacy requirement of methods refers to the routine recognition and production of local orders of social activities. As Garfinkel and Wieder (1992), (p. 184, our emphasis) put it, “ethnomethodology is concerned to locate and examine the concerted vulgar *uniquely adequate competencies* of order* production.”^10^ The enactment of methods of order production, or social practices, is uniquely adequate when the courses of action are recognizable for members and can be “taken seriously” by them (Garfinkel, 2022, p. 28)—or, as Hofstetter (2022) explains, “unique adequacy means being situated as some plausible local member.” It is a prerequisite for adequate analysis done by analysts both *lay and professional* (Garfinkel and Wieder, 1992, p. 183)—that is, not only by professional researchers (e.g., sociologists, ethnographers, or conversation analysts) but also by practitioners themselves in the studied settings, as they participate in concerted activities. Our earlier excursion into the development of telephony illustrates that as a competence in routine recognition and production of local order, unique adequacy has a historical dimension. For instance, what counts as adequate in landline telephony might not be adequate in mobile telephony. The skills for mundanely competent use of a technology, or *production* of social practice, may become obsolete, but they are still required for a *recognition* of that social practice in empirical materials from a former world, even if these practices are encountered as things of the past. Button et al. (2022, p. 75) point out that the methodological requirement of unique adequacy is “far from unique” to EM, being also incorporated in other disciplines, including the study of history (see, e.g., Simmel, 1907/1977; Kluback, 1956; Schwartz, 2017). Our proposal in this article moves toward a respecification of historical understanding as a practical recognition and production of potentially obsolescent practices, topicalizing “members’ reportable-observable production of the work itself” (Button et al., 2022, p. 75).

In preliminary studies of an early “chatbot” LYRIC in the late 1960s (see Eisenmann et al., forthcoming), working with printouts of interactions between the user and the machine, Garfinkel (1969, p. 3) noted “the difference between availability of ‘docile texts’ and texts available as a ‘first linear time through’ as contrasting phenomenal features of ‘conversing’ in man–machine conversations.” We understand this remark as proposing a distinction between lived sense-making work, embedded in a lifeworld, that goes on “in real time” and “*in situ*” when interacting with such a program (as “first linear time through”), and—on the other hand—the retrospective sense-making work involved in reading the transcript of user/machine interaction without a lifeworld correlate (as “docile texts”). This insight, highlighting the difference between the retrospective reading of a transcript and the lived experience of the situation, is also inspiring for considering EM/CA materials more broadly in the respecification of social change. Eventually, the recordings of practical actions and practical reasoning provided as EM/CA’s “data,” which made possible the transcripts with regard to landline and mobile telephony, can be read as texts that capture practices that are currently present or represent documents of a social history. Transcripts can be read as docile texts without a lifeworld correlate, or they can be explored as subtle details of lived historical practices by opening up the social embeddedness of interactional practices.

Our own analytical commentary above has been written in a way that highlights the historicity of practices, with the very comparison of telephone practices becoming the topic. Describing social activities, such as talking on the phone, requires a grasp of the sociomaterial reality in which they are done. Our analysis above supplies and enables such a grasp for a contemporary reader by providing contextual information that would not be necessary for an observer with a routine competence in the production of the described activities. Historically embedded practices are therefore made recognizable as meaningful actions for the readers, but the description alone does not allow for a proper reenactment (Sormani, 2016) of the interactional work. Sufficiently explained practices can make sense to observers, analysts, and readers of analytic accounts, even when these practices are not available anymore as something that they could themselves enact. Going through EM/CA’s corpus of studies, the historicity of members’ uniquely adequate competence is available as the encountered strangeness of everyday practices that are no longer accessible in their full, lived presence; these are practices that used to be taken for granted (e.g., opening a landline phone call) but have become obsolete and outdated, even while still being recognizable as meaningful for enactment of that practice. In the case of an obsolete practice, “mis-reading” the EM/CA descriptions as instructions (Garfinkel, 2002, p. 149) would be part of creating a member in a world of everyday praxis that no longer exists. Such considerations lead us to various possibilities for a respecification of social change as visible in captured details of routine practical action.

## 6. Multiple paths in the respecification of social change

EM/CA undertakes “a detailed study of social practices as a solution to the great theoretical problems of meaning and order” (Rawls, 2002, p. 3), which also include the classical theme of time and temporality (Rawls, 2005). So far, we have focused in this paper on arguing that EM/CA studies can be seen as a form of unintended, inadvertent, yet unavoidable social history. As a by-product of describing the here and now of a lived world, accounts of social praxis become historical accounts as the world they describe goes by. The intrinsic value of these analytic accounts rests in the fact that they describe social praxis ahistorically (i.e., without *a priori* consideration of historical development as part of the “context” in which it happens). We propose that this constitutes a first path for respecifying social change by a retrospective consideration of the corpus of EM/CA’s detailed studies of social activities as a resource to learn about obsolescent practices, such as the practices related to landline telephony, wayfinding with paper maps, or writing with a typewriter. This is related to focus on how “history gets done” in the temporality, sequentiality, and local historicity of social activities and their accumulative dimension (see Meyer and Schüttpelz, 2018, p. 196, in their discussion of Goodwin, 2018).

Moreover, in recent years, the historicity of social practices has been systematically examined in “longitudinal studies” in CA (Pekarek Doehler et al., 2018; Deppermann and Pekarek Doehler, 2021). In their introduction to the first edited collection of this line of research, Wagner et al. (2018) discuss two “pioneering studies on change over time”: Wootton’s work on the development of a child’s requests, and Clayman and Heritage’s research on changes in the organization of journalists’ questioning in presidential news conferences (see, e.g., Wootton, 1997; Clayman and Heritage, 2023). Our discussion of historical unique adequacy and practical obsolescence may bring further insights into this domain of study. We highlight the issue of recognizability (i.e., the routine visibility of the practice under consideration as a practice that is doing a particular action, such as *requesting* or *asking a question*), and the visibility of a practice as obsolescent. A practice is recognizable as achieving an action in a particular sociohistorical setting, and the routine recognizability of a practice in turn contributes to the constitution of just that “sociohistorical setting.” This is tied to the issue of comparability, and above all what constitutes a warrant for a “vertical comparison” (i.e., studying the development of practices; see Zimmermann, 1999). As proposed by Holland (1993/1978), p. 192, “We detect the sameness by seeing what persists within the constant change of our lives. We detect the difference by seeing what has changed against the background of sameness.” The practical ability to see social change in the details of everyday life is interwoven with the ability to see what remains unchanged, and to presuppose social structures such as individuals (e.g., who acquire conversational skills such as requesting) and institutions (e.g., within which speakers ask questions). Watson (2008, p. 210) points out that before being employed in professional analysis, comparison and contrasting are already members’ methods: “we can see ordinary interlocutors as ‘practical comparative sociologists’, making comparisons of categories or activities and working up contrasts on those bases.” This also leads to our final point.

**Figure 1:**
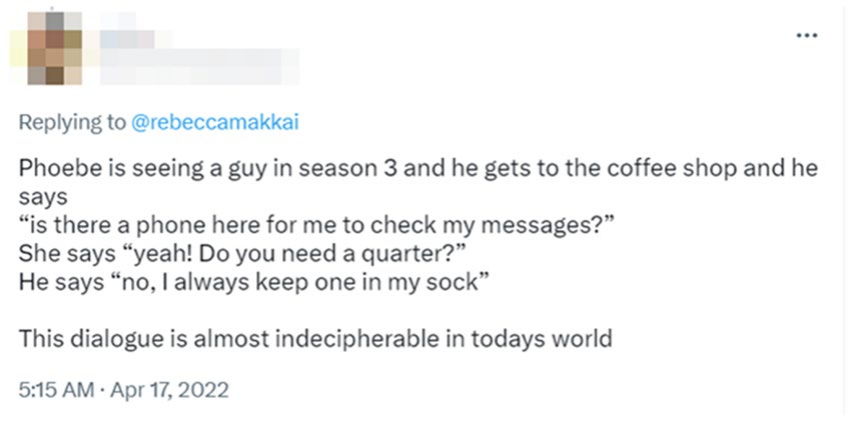
A comment on a Twitter post about the TV show Friends and some of the obsolescent practices it captures.

In order to identify and locate moments when the obsolescence of social practices becomes demonstrably consequential for the participants, one may also look at how members themselves orient to potentially obsolescent practices. This would allow us to investigate *emergent obsolescence* and capture the moments when previously commonplace practices are becoming obsolete, questionable, or disconnected from their sociohistorical environment. An example of the visible obsolescence of everyday practical knowledge could be a Twitter post by a mother who was (in the early 2020s) watching the TV series *Friends* (shot in the 1990s) with her daughter and had to explain many things that were taken for granted by the series creators but are not taken for granted any longer, including “what pagers were, and how they worked,” or “why secretaries answer office phones.”^11^ Many replies to the original post provide further material. Figure 1 is an illustrative instance.

An investigation of similar exchanges and accounts can provide an opportunity for a careful study of social change in the minutiae of everyday life, where new practices are discovered, invented, and sometimes praised, while old practices may be abandoned, problematized, and even ridiculed. EM/CA’s programmatic attention to detail (Garfinkel, 2022; Macbeth, 2022) may allow us to account *just how* these processes of social change occur in the lived interactional time of our everyday lives, while not being explicitly approached as “history” in the classical sense of a meaningful series of events and their disciplined study.

“History” in this classical sense can also become a subject of EM/CA’s deliberate focus, though it still holds true that “ethnomethodologists do not seem at home working on history” (Leudar and Nekvapil, 2011, p. 69). The major work on the local production of history remains Lynch and Bogen’s (1996) book on the Iran-Contra hearings, which shows how people establish, maintain, and contest “the past” in courtroom interactions. Following Lynch’s later suggestion to focus on “the practical and interactional production, reading, and establishment of documentary details” (2009, p. 98), Whittle and Wilson (2015, p. 58) turned their attention to the work of people tasked with “making history,” concluding that EM should aim at “explicating the practical actions (ethnomethods) through which versions of past events are worked up, worked on, and eventually ‘settled.’” Using CA, Burdelski (2016) analyzed stories of personal experiences of World War II in guided tours at a Japanese-American museum with regard to the narrators’ positioning as individuals and as collectivity members. He found that stories of personal experience told by docents are used as devices for identity construction, which encourages participation from visitors and helps achieve the educational goals of the visit. In these studies, history as a professional discipline becomes a topic of research, which is a related but tangential perspective *vis-à-vis* our aims in this article, where we instead emphasize the inherent historicity of all social life, and the possibility of its perhaps unexpected discovery in EM/CA studies that are radically focused on the here and now.

## 7. Concluding discussion

A text on “an archeology of the office” published by *The Economist* in October 2022 concludes: “Real archeologists need tools and time to do their painstaking work: paint brushes, trowels, sieves and picks. Corporate archeology is easier: you just need eyes and a memory of how things used to be. But you also need to be quick. As more and more workplaces are revamped for the hybrid era, now is the time to take a careful look around the office. You may see something that will soon seem as dated as pneumatic tubes, typewriters and fax machines.” Indeed, social practices that are technically mediated or augmented furnish us with highly illuminating topics, as they tend to undergo the most notable transformations, which occasionally can be swift and radical. In this article, we have suggested that as an aid for our “eyes and a memory of how things used to be,” one can revisit studies that were written as minute descriptions of an everyday world once present and taken for granted. Exploring the boundaries of sociological theory and ethnomethodology/conversation analysis (EM/CA), this invitation includes a shift in perspective by looking at EM/CA studies as a peculiar version of social history, in addition to their significance as studies of the structures of lived experience. Such a shift in perspective can be illuminating and worthwhile for scholars in social sciences more generally, as well as for researchers who conduct EM/CA inquiries themselves.

When we look at the wide spectrum of existing EM/CA studies, we can get a sense of the potential of EM/CA as a discipline dealing with history and social change. There has been a lively tradition of studies on “institutional interaction,” which will soon reveal many ways of how things were once done (Arminen, 2017). There is a long list of institutions that have undergone profound changes in past decades, from control rooms to police work, and from offices to classrooms. Numerous institutional practices have been captured and analyzed by EM/CA researchers. The circumstantial lived detail of social activities examined in EM/CA is undergoing rapid transformations—when offices become paperless, police officers carry cameras, control and technical support rooms are transported to other continents, and students are provided with digital tools. As an outcome of such processes of social change—more or less technologized—we have a plethora of thorough and systematic studies of practices that are no longer practiced. Inadvertently, EM/CA studies also capture cultural changes: past civil politeness toward politicians, explicit assumptions of gender roles occupied by husbands and wives, or AIDS therapy from a time when there was not yet HIV.^12^ Finally, recent EM/CA studies of new practices established during the COVID-19 pandemic have also captured a historical reality, as many of these practices (e.g., greeting with elbow bumps; see Mondada et al., 2020) may have already become a thing of the past, since the lifeworld in which these practices were meaningful is no longer there.

In this context, our paper has considered the notion of obsolescence of social practices as a way to gain access to the inherent historicity of social life, while at the same time praxiologically respecifying the fundamental sociological topic of social change. Further work in this direction could investigate whether there are different kinds of obsolescence, as one could expect that the obsolescence of a social practice might range from marginalization and disappearance to total incomprehensibility. One may see a particular action (e.g., a greeting or a request) done in an obsolescent fashion while still recognizing it as that action, or one may see past conduct that is void of any meaning, having become completely obsolete. As a whole, were EM/CA able to articulate a path from the emergence of new social practices to their routinization and habituation, it would capture glimpses of the historicity of human agency, which is beautifully propounded by Ernaux (2008/2022), p. 205: “The questions that arose with the appearance of new technologies were canceled out as their use became second nature, and required no thought. People who did not know how to use a computer or a Discman would become obsolete, like those who could not use a phone or washing machine.” The skilled ability to use a technological object in a routine, mundane, unremarkable way is related to the uniquely adequate competences that comprise the practical accomplishment of professional and everyday activities, such as talking on the phone, following a map, or doing laundry. As we discussed above, the unique adequacy requirement of methods has a historical dimension that must be considered in specifying the complex relations between members’ practical knowledge and the possibility of its recovery from analytical accounts and descriptions.

The historical perspective that accentuates social change poses certain challenges for EM/CA studies. As mentioned, much of the EM/CA research concerns history and social change only inadvertently and under a particular reading. Researchers may have a fine-grained sophisticated grasp of the subtle nuances of interactional practices but possess only limited resources to reflect the linkage of social interaction to the passage of sociohistorical “Big Time” (Button, 1990). The notion of obsolescence may provide solutions and insights related to some general challenges in “longitudinal CA” (Pekarek Doehler et al., 2018; Deppermann and Pekarek Doehler, 2021), such as the partial nature of the data, the comparability of phenomena across collections from different time periods, and issues in documenting and explaining change in social practices. Taking into account the obsolescence of practices as a members’ phenomenon repositions these methodological issues as topics grounded in the historical particularity of the examined social activities, putting forward the encounters with social change and “vertical comparison” as something that participants themselves deal with. Ultimately, respecifying social change means that we also must respecify what we consider to be “history,” or historically relevant, or historically constituted. When we return to Sacks (1992, Vol. I, p. 94) contemplation of the historicity of practices*—*“A thing that our forefathers had. Like God”—we may also read it as related to the familiar sociological thesis of secularization. Were we to recover and respecify the sense of history and social change available or assumed in the studies that have already been done in EM/CA, we would not run out of work too soon.

The classical sociological tradition of studying social change was burdened with troubles. Although it was able to portray nuanced degrees of social evolution, “the concrete contours” and “crystallizations” of change remained undetermined, and scholars were restricted to “indicate ranges of possibilities” (Eisenstadt, 1964, p. 386). Later, narrower meso-level approaches, such as domestication of media and technologies (e.g., Sørensen, 2006), enabled a finer grasp of emerging social practices. As Sørensen (2006, p. 55) summarizes “the impact of mobile telephony”: “What is new is that one should be accessible everywhere and at all times.” Domestication is a metaphor of taming the beast, making it known, familiar, stable, and docile. As such, the perspective catches the meso-level social change, but it risks losing the radical aspects of change. When people appropriate new practices, they do not just tame artifacts and technologies, but also make previous practices and identities obsolete. EM/CA may retain sensitivity to emerging new practices as it studies the ways in which people make relevant objects and artifacts for their actions, which may, however, appear as if the technologies themselves (e.g., landline and mobile phone) featuring in the formation of action had vanished (see Button, 1993). Technical features tend to become oriented to by the participants only when there is a problem, when something fails to work, and parties reorient to find out what to do next, or reason about the nature of the problem to get around it or repair it (Kosurko et al., 2023; Mlynář et al., 2023; Tiilikainen et al., 2023). And even when there is a technical problem, it is not self-evident that interactants treat the problem as a problem, as they may make use of it, and utilize the “problem” for their own purposes (Rintel, 2013). Therefore, the monocausal versions of technological determinism seem to fail (see Ogburn, 1947; Wyatt, 2008).

The inevitable counterpart of obsolescence is persistence, offering a complementary perspective of focusing on the emergence of new practices. As soon as EM/CA findings are somehow connected to sociohistorical reality, the analyst is bound to take stances; if the analysis is completely detached from the sociohistorical world, it remains purely technical. By looking at telephony, and technologized interaction more generally, we have intentionally prioritized change over stability for the purposes of illustration. Indeed, many social practices—if they ever become truly obsolete—remain remarkably stable over time. To stress the salience of “obsolescence,” we have not yet discussed variabilities of “obsolescences” or their degrees, not to mention the closely related topics of perseverance of social practices, or the appearance of novel and innovative ones. Throughout, nevertheless, we have argued that such questions should, first and foremost, be answered empirically. If our paper provides inspiration for a further respecification of social change in the sense discussed above, then its purpose has been fulfilled.

## Ethics statement

Ethical approval was not required for the study involving human data in accordance with the local legislation and institutional requirements. Written informed consent was not required for participation in the study or for the publication of potentially identifiable data in accordance with the national legislation and the institutional requirements. The social media data was accessed and analyzed in accordance with the platform’s terms of use.

## Author contributions

All authors contributed equally to the writing of the manuscript, while the idea behind it was proposed by JM as initial grounds for the collaboration.

## Figures and Tables

**Table 1:** 

[]	Overlapping talk.
(.)	Micro-pause.
(2.2)	Pause in seconds.
.	Final intonation.
>yes<	Notably faster talk.
	Notably slower talk.
(but)	Estimated hearing.
()	Inaudible segment.
a::	Vocal prolongation.
Re-	Cut-off.
↑	Higher pitch.
=	Rapid continuation (latching).
.hh/hh	Inhalation and exhalation.
n(h)o	Laughter particle within word.
THAT	Louder volume.
that	Hearable emphasis.

## Footnotes

^1^WWR stands for *Walking with Romans*, a digital “guide app … developed to facilitate physical and historical access to a little-visited site” (Smith et al., 2020, p. 226).

^2^EM and CA are approaches that have developed in sociology of the 1950s and 1960s, mainly in the work of Garfinkel and Sacks (Sacks, 1963, 1972, 1992; Garfinkel, 1967, 2002, 2022; Garfinkel and Sacks, 1970). The aim of EM/CA is to describe and explicate the systematic ways by which people produce orderly events and actions in social interaction. It takes as its distinctive phenomenon members’ methods of practical reasoning and practical action in everyday and specialized social settings (Livingston, 1987; Lynch, 1993), and the organization of talk-in-interaction (Schegloff, 2007). For more recent developments, see, e.g., Button et al., 2022; Maynard and Heritage, 2022; Haddington et al., 2023; Sormani and vom Lehn, 2023.

^3^In spy movies, or when a Westerner was staying in an Eastern-bloc hotel, one would listen immediately if the line-opening sound was accompanied with a silent click.

^4^Over the course of the history of telephony, the network grew denser as the number of points increased. Praxiologically, were we to explore this aspect of archived recordings, this would also have a correlate in telephone communication practices.

^5^In classic comedy films, there are several scenes of a call recipient believing they know who is calling already on the basis of the summons, leading to numerous comical developments due to a presupposed caller identification.

^6^Were we to critically study classic comedies (note 5 above), we might note that some of the types of confusion would not have happened had the receiver opened the line with the super-confident style, revealing that they had presumed to know who had called. Alternatively, the receiver may have intentionally hidden their presupposition of the callers’ identity. This scenario would set up these comedy scenes for “strategic interaction,” where the actors’ try to hide aspects of their knowledge due to strategic reasons (Goffman, 1969). A wider point here is that historically varying epistemic ecosystems open up different action possibilities, including for “strategic” actions.

^7^In an ideal world, we could next show how “reporting” has become reconfigured with mobile technologies. Indeed, reporting practices are common in mobile telephony: people both “report” their whereabouts and are also held responsible for that over the phone (Arminen, 2006; Arminen and Leinonen, 2006). In a way, Excerpt 2 also develops that direction. We do not really dwell on that aspect. In reality, we have a limited number of pre-teenagers’ calls that offered relevant material for a strict comparison of the reporting practices in question. We must return to the emerging reporting practices on another occasion.

^8^Arminen and Leinonen (2006) have extended the discussion of “no” and its untranslatability; see, in particular, note 5. Other studies of “no” include Sorjonen and Vepsäläinen (2016) and Vepsäläinen (2019).

^9^Similarly to how self-driving cars are capable of traveling without human activity, digital telephone systems identify the caller without human input. While self-driving cars are still in a test phase, digital telephone systems have been in operation for some time.

^10^Regarding “order” spelled with an asterisk, an endnote explains: “Spelled with an asterisk, order* is a collector and a proxy for any and every topic of logic, meaning, method, reason, and order. It stands in for any and all the marvelous topics that are available in received lingoes and received topics in intellectual history. Of course these include the lingoes and studies in the endless arts and sciences of practical action” (Garfinkel and Wieder, 1992, p. 202).

^11^Available online at: https://twitter.com/rebeccamakkai/status/1515467051959304193 (accessed 10. 8. 2023).

^12^The notion of HIV (human immunodeficiency virus) was formulated later than AIDS (acquired immunodeficiency syndrome). Therefore, there was “AIDS” before “HIV” was discovered.

## Appendix

Transcription conventions (based on Jefferson, 2004).
